# Syndesmotic instability can be assessed by measuring the distance between the tibia and the fibula using an ultrasound without stress: a cadaver study

**DOI:** 10.1186/s12891-022-05221-z

**Published:** 2022-03-18

**Authors:** Hiroaki Shoji, Atsushi Teramoto, Yasutaka Murahashi, Kota Watanabe, Toshihiko Yamashita

**Affiliations:** 1 Department of Orthopaedic Surgery, Sapporo Medical University School of Medicine, South-1, West-16, Chuo-ku, Sapporo, 060-8543 Japan; 2 Second Division of Physical Therapy, Sapporo Medical University School of Health Sciences, South- 1, West-16, Chuo-ku, Sapporo, 060-8543 Japan

**Keywords:** Syndesmosis injury, Ultrasound, Tibiofibular distance, Cadaver, Stress load

## Abstract

**Background:**

Ultrasound examinations for syndesmosis injury might be useful for the quantitative evaluation of syndesmotic instability. The purpose of this study was to evaluate the efficacy of ultrasound assessment by measuring the tibiofibular distance of syndesmosis injuries in various ligament-injured models and stress load conditions.

**Methods:**

Five normal ankles from Thiel-embalmed cadavers were used. Ultrasound assessment was performed by placing a probe in parallel with the ligament running just above the anterior inferior tibiofibular ligament (AITFL). The distance between the anterior border of the tibia and the fibula was measured in the intact condition. Next, Bassett’s ligament was cut arthroscopically to reduce damage to soft tissues as much as possible and measurement was performed in the same way. After that, the AITFL, interosseous membrane (IOM), deltoid ligament, and posterior inferior tibiofibular ligament (PITFL) were macroscopically cut and measured in that order. Ankle positions were without stress (natural plantar flexion without applying stress to the ankle joint), dorsiflexion stress, inversion stress, and external rotation stress. All stress to the ankle joint was carried out manually to the maximum extent.

**Results:**

As with the without-stress condition, significant increases in tibiofibular distances after AITFL dissection were seen compared with the intact state under all stress conditions (intact: 4.9 ± 1.0 mm without stress, 5.6 ± 1.2 mm with dorsiflexion, 5.9 ± 1.0 mm with inversion, and 6.7 ± 1.3 mm with external rotation; AITFL dissection: 6.7 ± 1.5 mm without stress, 7.3 ± 1.2 mm with dorsiflexion, 7.5 ± 1.4 mm with inversion, and 8.7 ± 1.6 mm with external rotation). AITFL dissection with external rotation stress significantly increased the tibiofibular distance compared to without stress.

**Conclusion:**

Changes in tibiofibular distance with the severity of syndesmosis injury were measured by ultrasound using cadavers. No significant change was seen with Bassett’s ligament injury, but tibiofibular distance increased significantly with injuries of equal to or greater severity than AITFL injury. Results were similar not only for external rotation stress, but also for dorsiflexion stress and inversion stress, and even in unloaded states, significant tibiofibular widening was confirmed with injuries of equal to or greater severity than AITFL injury.

## Introduction

Syndesmosis injuries mainly occur simultaneously with ankle fractures, but sometimes occur without fractures. Independent syndesmosis injuries have been reported to occur in 17–74% of ankle injuries, representing considerable variation between reports [1–3]. This might be attributable to the low diagnostic rate. Previous studies have reported a diagnostic rate of 63–71% with plain radiography and 54–67% with manual stress tests [4, 5]. In addition, manual stress tests are often difficult to perform due to swelling and pain in the affected region during the acute phase after injury. If syndesmosis injury remains undiagnosed, chronic pain or instability can result and lead to osteoarthritis of the ankle [6, 7]. Improvements in the diagnostic rate are therefore necessary.

Ultrasound (US) shows the following advantages: easy to perform in outpatient and bedside settings; minimal invasiveness without radiation exposure; possibility of dynamic evaluation; assistance with injection; and quantitative evaluation. Given these benefits, this modality has been popular in the field of orthopedics in recent years. Regarding US for the ankle joint, although some reports have described the use of US for the anterior talofibular ligament (ATFL) of the ankle joint, few have reported on its use in syndesmosis injuries [8–10]. Hagemeijer et al. showed syndesmosis motion under stress condition by US [11]. But this study was only for healthy participants without ankle injuries. Lee et al. reported that US examination showed high sensitivity and specificity for syndesmosis injury [12]. However, that investigation only used US for qualitative diagnosis of anterior inferior tibiofibular ligament (AITFL) injury, and evaluation of the syndesmotic instability was not performed. Since quantitative evaluations can be performed with US, syndesmotic instability could be evaluated by measuring the distance between the tibia and fibula. Evaluation under loading with external rotation stress has been reported, but results under other stresses remain unknown [13]. In addition, applying stress loads to acute syndesmosis injuries remains unsafe because of the invasiveness to patients.

The aim of this study was to evaluate the efficacy of US assessment for diagnosing syndesmosis injuries in various ligament injuries and under different stress load conditions. The hypothesis for this study was that syndesmotic instability can be assessed by measuring the distance between the tibia and fibula using US without stress.

## Materials and methods

This study was approved by the ethics committee of our institution. Five normal ankles from Thiel-embalmed cadavers (2 male, 3 female) that had been donated to the department of anatomy of our university were used. Mean age at time of death was 82.4 years (range, 73–92 years). After embalming the cadavers with Thiel’s method [14], the whole body was stored with the entire legs. Cadavers with obvious deformity of the ankles or past surgical history involving the ankles were excluded. After all ligament transection and testing, a macroscopic check was performed to ensure that no degenerative changes were apparent in the ankle joint.

**Fig. 1:**
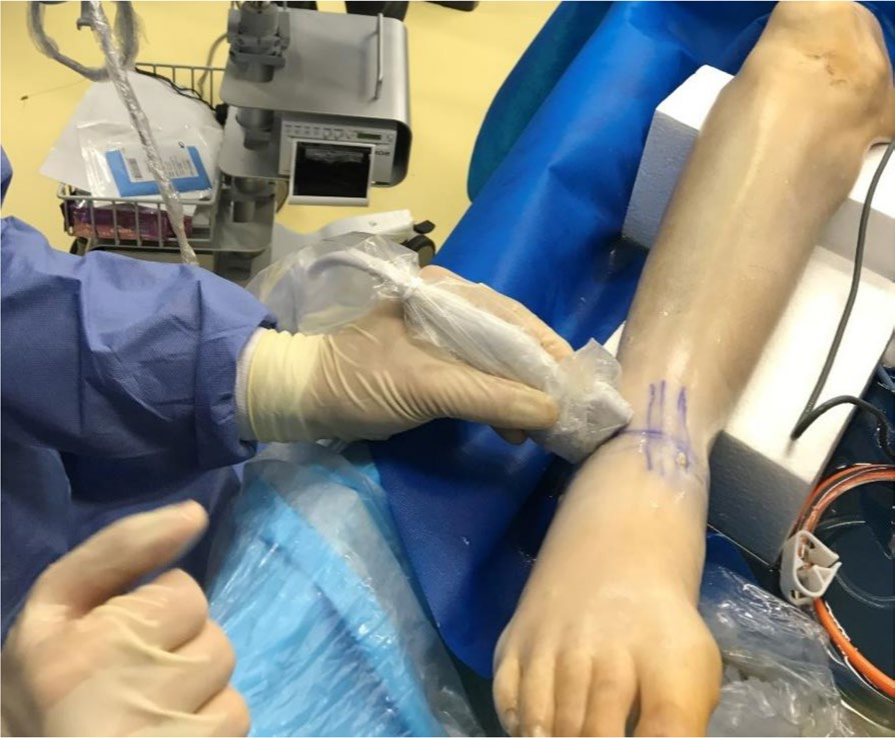
Placement of the ultrasound probe in parallel with the ligament running just above the AITFL, 30 degrees from the ankle joint

**Fig. 2:**
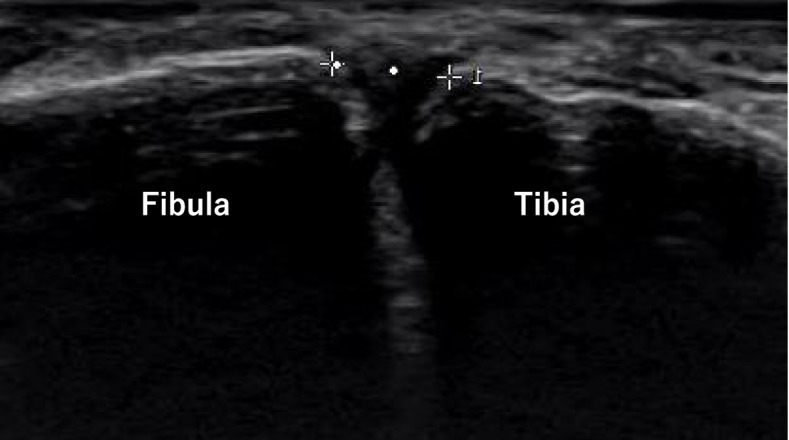
Distance between the anterior border of the tibia and the fibula was measured

All US assessments were performed by two orthopedic surgeons. All experiments were performed once by each surgeon. A SONOIMAGE HS 1 system (Konica Minolta Japan Inc., Tokyo, Japan) with an L18-4 linear probe at 10 MHz in B-mode was used for US assessment. In US assessments, the AITFL was visualized by placing a probe in parallel with the ligament running just above the AITFL at first (Fig. 1). Next, the distance between the anterior border of the tibia and the fibula was measured in that position (Fig. 2).

Following the assessments with the ankles in the intact condition, a series of similar assessments with the syndesmosis injury models began with a sectioned Bassett’s ligament (as the accessory anteroinferior tibiofibular ligament [15]), followed by a sectioned AITFL, then with a sectioned interosseous membrane (IOM) distal 15 cm ligament, and finally with the deltoid ligament and posterior inferior tibiofibular ligament (PITFL) both sectioned. We created a model of serious injuries from minor injuries in stages [16]. Serious syndesmosis injuries are often associated with deltoid ligament injuries [17]. In addition, the frequency of PITFL rupture was extremely low [18]. So, we cut the ligaments in this order. Bassett’s ligament was cut arthroscopically to reduce damage to soft tissues as much as possible, and the other ligaments were cut macroscopically in separate small incisions from the arthroscopic portal using a knife. A 2.7-mm, 30°, oblique arthroscope was inserted via the antero-medial portal, and a shaver was inserted via the antero-lateral portal. All specimens had Bassett’s ligament and were cut by shaver.

The specimen was not cut, and whole body was positioned supine. Ankle positions were natural plantar flexion without applying stress to the ankle joint (without stress), dorsiflexion stress, inversion stress, and external rotation stress position. All stress to the ankle joint was carried out manually to the maximum extent. The maximum extent depended on the competence of each surgeon. Stress tests and ultrasound scanning were performed by the same surgeon at the same time. This reproduced the actual clinical situation.

### Data analysis

Measurements were made by the same two orthopedic surgeons and mean values were used for analysis. We performed this measurement method in advance in the 8 feet of 4 healthy volunteers. Intraobserver reliability was evaluated using the intraclass correlation coefficient (ICC) between two readings, and the ICC for interobserver reliability was determined by comparing measurements by the two orthopedic surgeons. All data were analyzed using statistical software (EZR; Saitama Medical Center, Jichi Medical University, Saitama, Japan), which is a graphical user interface for R (The R Foundation for Statistical Computing, version 2.13.0). First, the normality of all outcome data was confirmed using the Shapiro–Wilk test. Since all outcome data were in accordance with normal distributions, one-way repeated-measures analysis of variance was used for comparisons across each group. Post-hoc analyses for differences among parameters were checked using Dunnett’s test. Values of *P* < 0.05 were considered significant.

## Results

### Measurement accuracy of US assessment

Intraobserver ICC was 0.97 (95% CI: 0.90–0.99) and interobserver ICC was 0.85 (95% CI: 0.71–0.92) in the assessments of healthy volunteers.

### Tibiofibular distance without stress

Tibiofibular distances in natural plantar flexion without stress are shown in Table 1. Although no significant increase was seen with dissection of Bassett’s ligament, tibiofibular distances were significantly larger after AITFL dissection compared with the intact state.

**Table 1:** Tibiofibular distances in natural plantar flexion without stress

	Tibiofibular distance	
Ligament sectioned	(mean ± SD, mm)	*P*
Intact	4.9 ± 1.0	-
Bassett’s	5.5 ± 1.5	0.796
AITFL	6.7 ± 1.5	0.022*
IOM	7.4 ± 1.1	0.001*
Deltoid	7.5 ± 0.7	< 0.001*
PITFL	8.4 ± 1.4	< 0.001*

*AITFL* anterior inferior tibiofibular ligament, *IOM* interosseous membrane, *PITFL* posterior inferior tibiofibular ligament, *SD* standard deviation

^*^Significant difference compared with the intact model (*P* < 0.05)

### Tibiofibular distance with stress

Tibiofibular distances under dorsiflexion stress, inversion stress, and external rotation stress are shown in Tables 2, 3, and 4, respectively. As in the without-stress condition, no significant increase was seen with dissection of Bassett’s ligament and significant increases were seen after AITFL dissection compared with the intact state under all stress conditions.

**Table 2:** Tibiofibular distances under dorsiflexion stress

	Tibiofibular distance	
Ligaments sectioned	(mean ± SD, mm)	*P*
Intact	5.6 ± 1.2	-
Bassett’s	5.9 ± 1.4	0.991
AITFL	7.3 ± 1.2	0.028*
IOM	8.3 ± 1.0	< 0.001*
Deltoid	8.6 ± 0.9	< 0.001*
PITFL	9.6 ± 1.2	< 0.001*

*AITFL* anterior inferior tibiofibular ligament, *IOM* interosseous membrane, *PITFL* posterior inferior tibiofibular ligament, SD, standard deviation

^*^Significant difference compared with the intact model (*P* < 0.05)

**Table 3:** Tibiofibular distances under inversion stress

	Tibiofibular distance	
Ligaments sectioned	(mean ± SD, mm)	*P*
Intact	5.9 ± 1.0	-
Bassett’s	6.4 ± 1.5	0.827
AITFL	7.5 ± 1.4	0.031*
IOM	8.9 ± 1.0	< 0.001*
Deltoid	8.7 ± 0.8	< 0.001*
PITFL	9.6 ± 1.2	< 0.001*

*AITFL* anterior inferior tibiofibular ligament, *IOM* interosseous membrane, *PITFL* posterior inferior tibiofibular ligament, *SD* standard deviation

^*^Significant difference compared with the intact model (*P* < 0.05)

**Table 4:** Tibiofibular distances under external rotation stress

	Tibiofibular distance	
Ligaments sectioned	(mean ± SD, mm)	*P*
Intact	6.7 ± 1.3	-
Bassett’s	7.1 ± 1.7	0.956
AITFL	8.7 ± 1.6	0.015*
IOM	9.5 ± 0.9	< 0.001*
Deltoid	9.5 ± 0.9	< 0.001*
PITFL	9.9 ± 1.1	< 0.001*

*AITFL* anterior inferior tibiofibular ligament, *IOM* interosseous membrane, *PITFL* posterior inferior tibiofibular ligament, *SD* standard deviation

^*^Significant difference compared with the intact model (*P* < 0.05)

## Discussion

This study investigated tibiofibular distance using US in cadaveric models. The results showed that although no significant increases were seen with dissection of Bassett’s ligament, tibiofibular distances were significantly larger after AITFL dissection compared with the intact state in any stress or without stress conditions. We got these results without stress condition, invasive loading appears avoidable in cases of acute injury, and diagnosis can be reached by comparison of both ankles in conditions without stress. These were as we hypothesized.

Diagnostic imaging of syndesmosis injury is often performed by magnetic resonance imaging (MRI). MRI can firmly evaluate signal changes associated with damage to the AITFL and interosseous membrane [19]. MRI is also useful because of the ability to diagnose diastasis and subluxation of the tibiofibular joint [19]. On the other hand, false positives can be seen for AITFL injury due to mismatch between AITFL runs and MRI slices [20]. Computed tomography (CT) is used for evaluating widening between the tibia and fibula in greater detail. CT is useful for identifying subluxation of the tibiofibular joint and evaluating reduction after treatment. Since syndesmosis injury is often accompanied by fractures, fracture evaluation can be performed at the same time. The positional relationship of the fibula to the tibia varies by individual, and even in a normal ankle joint, false widening may be misestimated if the distance is large. CT images should be evaluated with the inclusion of both feet in the same image [21]. In recent years, CT imaging under loaded conditions has become possible [22]. Since the fibula moves physiologically depending on the load, weight-bearing CT offers more realistic evaluated and greater reliability. In cases where surgery is necessary, arthroscopy allows evaluation of the widening of the tibiofibular joint [23]. However, this evaluation is limited to surgical cases, and not all cases are targeted because it is an invasive technique with related risks.

The application of US to syndesmosis injuries has been reported as useful in athletes [24]. This modality allows evaluation of dynamic movements of the fibula, and physiological fibular movements can be evaluated by US even in the normal ankle [11]. As for the evaluation of syndesmosis instability, a method of measuring tibiofibular distance by US has been reported (as applied in this study) [13]. In that cadaveric experiment, distance between the tibia and fibula was quantitatively evaluated by adding external rotation stress. In our study, we modeled more detailed injury conditions involving structures such as Bassett’s ligament and interosseous membranes, in addition to states without stress and under external rotation stress. Tibiofibular distance was measured by US with the addition of dorsiflexion and inversion stress. Since the diastasis between the tibia and fibula was found to be significantly increased with injuries of equal to or greater severity than AITFL injury without external rotation stress, invasive loading appears avoidable in cases of acute injury, and diagnosis can be reached by comparison of both ankles in conditions without stress. Since external rotation stress significantly increases the tibiofibular distance compared to the condition without stress, measurement of diastasis by ultrasound may be better with the addition of external rotation stress in chronic cases.

Syndesmosis injury is not easy to diagnose and therapeutic effects can be difficult to judge. Multiple MRI scans after treatment has the problem of excessive medical costs, while radiation exposure is a problem for CT. US has no such problems and is easily used as a diagnostic tool. Adding the same external rotation stress is difficult when comparing situations before and after treatment in clinical settings. However, since diastasis was found to change with AITFL injury by US with or without stress, measurement of the tibiofibular distance by US under conditions without loading may be an effective method of evaluation.

This study has the following limitations. First, measurements were made in cadavers. Since measurements in injury models were made by cutting ligaments with a blade, numerical values may differ from those in actual injured patients. In addition, since Thiel-embalmed cadavers were used, the joints may have shown laxity or hardening compared to physiological conditions. Second, the number of specimens and measurements was small. Although the study was conducted using an evaluation method with a high ICC, reliability and validity would both be enhanced by performing more evaluations. Third, all stress conditions were applied manually. The lack of constant loads may have affected the results. By applying a larger load, a significant difference may have been created compared to the intact state even under conditions other than external rotation stress. However, applying a constant load is difficult in actual clinical settings, and measurement result under load applied manually while using the US probe were considered more realistic. Despite these limitations, this study showed changes in tibiofibular distance according to the severity of syndesmosis injury by US measurement. As a result of examining not only external rotation stress, but also unloaded conditions, we were able to identify an increase in the distance between the tibia and fibula in AITFL injury. The present results will be clinically applicable to the diagnosis of syndesmosis injuries, and judgment of both severity and therapeutic efficacy.

## Conclusion

The tibiofibular distance changes with the severity of syndesmosis injury as measured by US in cadavers. No significant change was seen with Bassett’s ligament injury, but tibiofibular distance was significantly increased with injuries of equal to or greater severity than AITFL injury. The result was the same not only for external rotation stress, but also for dorsiflexion stress and inversion stress, and even in the state without load, significant tibiofibular widening was confirmed with injuries of equal to or greater severity than AITFL injury.

## Data Availability

The datasets generated and/or analyzed during the current study are not publicly available due to continuing research using this data, but are available from the corresponding author on reasonable request.

## References

[1] Boytim MJ, Fischer DA, Neumann L (1991). Syndesmotic ankle sprains. Am J Sports Med.

[2] Gerber JP, Williams GN, Scoville CR, Arciero RA, Taylor DC (1998). Persistent disability associated with ankle sprains: a prospective examination of an athletic population. Foot Ankle Int.

[3] Wright RW, Barile RJ, Surprenant DA, Matava MJ (2004). Ankle syndesmosis sprains in National Hockey League players. Am J Sports Med.

[4] Takao M, Ochi M, Oae K, Naito K, Uchio Y (2003). Diagnosis of a tear of the tibiofibular syndesmosis. The role of arthroscopy of the ankle. J Bone Joint Surg Br.

[5] Sman AD, Hiller CE, Rae K, Linklater J, Black DA, Nicholson LL, Burns J, Refshauge KM (2015). Diagnostic accuracy of clinical tests for ankle syndesmosis injury. Br J Sports Med.

[6] Leeds HC, Ehrlich MG (1984). Instability of the distal tibiofibular syndesmosis after bimalleolar and trimalleolar ankle fractures. J Bone Joint Surg Am.

[7] Nussbaum ED, Hosea TM, Sieler SD, Incremona BR, Kessler DE (2001). Prospective evaluation of syndesmotic ankle sprains without diastasis. Am J Sports Med.

[8] Lee KT, Park YU, Jegal H, Park JW, Choi JP, Kim JS (2014). New method of diagnosis for chronic ankle instability: comparison of manual anterior drawer test, stress radiography and stress ultrasound. Knee Surg Sports Traumatol Arthrosc.

[9] Cho JH, Lee DH, Song HK, Bang JY, Lee KT, Park YU (2016). Value of stress ultrasound for the diagnosis of chronic ankle instability compared to manual anterior drawer test, stress radiography, magnetic resonance imaging, and arthroscopy. Knee Surg Sports Traumatol Arthrosc.

[10] Kemmochi M, Sasaki S, Fujisaki K, Oguri Y, Kotani A, Ichimura S (2016). A new classification of anterior talofibular ligament injuries based on ultrasonography findings. J Orthop Sci.

[11] Hagemeijer NC, Saengsin J, Chang SH, Waryasz GR, Kerkhoffs GMMJ, Guss D, DiGiovanni CW (2020). Diagnosing syndesmotic instability with dynamic ultrasound - establishing the natural variations in normal motion. Injury.

[12] Lee SH, Yun SJ (2017). The feasibility of point-of-care ankle ultrasound examination in patients with recurrent ankle sprain and chronic ankle instability: Comparison with magnetic resonance imaging. Injury.

[13] Fisher CL, Rabbani T, Johnson K, Reeves R, Wood A (2019). Diagnostic capability of dynamic ultrasound evaluation of supination-external rotation ankle injuries: a cadaveric study. BMC Musculoskelet Disord.

[14] Thiel W (1992). The preservation of the whole corpse with natural color. Ann Anat.

[15] Bassett FH, Gates HS, Billys JB, Morris HB, Nikolaou PK (1990). Talar impingement by the anteroinferior tibiofibular ligament. A cause of chronic pain in the ankle after inversion sprain. J Bone Joint Surg Am..

[16] Shoji H, Teramoto A, Suzuki D, Okada Y, Sakakibara Y, Matsumura T, Suzuki T, Watanabe K, Yamashita T (2018). Suture-button fixation and anterior inferior tibiofibular ligament augmentation with suture-tape for syndesmosis injury: A biomechanical cadaveric study. Clin Biomech (Bristol, Avon).

[17] Miller CD, Shelton WR, Barrett GR, Savoie FH, Dukes AD (1995). Deltoid and syndesmosis ligament injury of the ankle without fracture. Am J Sports Med.

[18] Warner SJ, Garner MR, Schottel PC, Hinds RM, Loftus ML, Lorich DG (2015). Analysis of PITFL injuries in rotationally unstable ankle fractures. Foot Ankle Int.

[19] Sharif B, Welck M, Saifuddin A (2020). MRI of the distal tibiofibular joint. Skeletal Radiol.

[20] Oae K, Takao M, Naito K, Uchio Y, Kono T, Ishida J, Ochi M (2003). Injury of the tibiofibular syndesmosis: value of MR imaging for diagnosis. Radiology.

[21] Kubik JF, Rollick NC, Bear J, Diamond O, Nguyen JT, Kleeblad LJ, Helfet DL, Wellman DS (2021). Assessment of malreduction standards for the syndesmosis in bilateral CT scans of uninjured ankles. Bone Joint J.

[22] Hagemeijer NC, Chang SH, Abdelaziz ME, Casey JC, Waryasz GR, Guss D, DiGiovanni CW (2019). Range of Normal and Abnormal Syndesmotic Measurements Using Weightbearing CT. Foot Ankle Int.

[23] Teramoto A, Shoji H, Anzai K, Kamiya T, Watanabe K, Yamashita T (2020). Tibiofibular Space Widening Assessment with a Ball-Tipped Probe in a Syndesmosis Injury Model. J Foot Ankle Surg.

[24] Mei-Dan O, Kots E, Barchilon V, Massarwe S, Nyska M, Mann G (2009). A dynamic ultrasound examination for the diagnosis of ankle syndesmotic injury in professional athletes: a preliminary study. Am J Sports Med.

